# National Trends in Prevalence of Depression in Men and Women with Chronic Obstructive Pulmonary Disease Hospitalized in Spain, 2016–2020

**DOI:** 10.3390/jcm11216337

**Published:** 2022-10-27

**Authors:** Javier de Miguel-Díez, Ana Lopez-de-Andres, Rodrigo Jimenez-Garcia, Jose M. de Miguel-Yanes, Valentin Hernández-Barrera, David Carabantes-Alarcon, Jose J. Zamorano-Leon, Concepcion Noriega

**Affiliations:** 1 Respiratory Department, Hospital General Universitario Gregorio Marañón, Facultad de Medicina, Instituto de Investigación Sanitaria Gregorio Marañón (IiSGM), Universidad Complutense de Madrid, 28009 Madrid, Spain; 2 Department of Public Health and Maternal & Child Health, Faculty of Medicine, Universidad Complutense de Madrid, 28040 Madrid, Spain; 3 Internal Medicine Department, Hospital General Universitario Gregorio Marañón, Instituto de Investigación Sanitaria Gregorio Marañón (IiSGM), Universidad Complutense de Madrid, 28009 Madrid, Spain; 4 Preventive Medicine and Public Health Teaching and Research Unit, Health Sciences Faculty, Universidad Rey Juan Carlos, 28922 Alcorcón, Spain; 5 Department of Nursery and Physiotherapy, Faculty of Medicine and Health Sciences, University of Alcalá, 28801 Alcalá de Henares, Spain

**Keywords:** COPD, depression, sex, prevalence, in-hospital mortality, trends, Spain

## Abstract

(1) Background: To describe trends in the prevalence of depression in men and women with COPD hospitalized in Spain (2016–2020). (2) Methods: We used a nationwide discharge database to select all patients ≥35 years with COPD. (3) Results: The prevalence of depression was 3.54-times higher in women with COPD than in men (OR 3.54; 95%CI 3.48–3.6). It decreased significantly between 2016 and 2020, although the reduction was only significant in women (12.27% in 2016 vs. 10.56% in 2020). Older age, comorbidity and the most recent years of hospital admission were associated with lower prevalence of depression in both men and women, while obesity, obstructive sleep apnea (OSA) and use of oxygen prior to admission were risk factors. In-hospital mortality (IHM) increased significantly over time. Older age, comorbidity, the use of oxygen prior to admission and having been hospitalized in 2020 increased the risk of IHM. Female sex was associated with a lower IHM in patients with depression and COPD. (4) Conclusions: The prevalence of depression has decreased over time in women with COPD while it has not changed significantly in men with this disease. IHM increased over time both in men and women with COPD and depression, with higher prevalence in the former.

## 1. Introduction

Chronic obstructive pulmonary disease (COPD) is a common clinical condition that is associated with significant worldwide morbidity and mortality [1]. It is increasingly recognized that comorbidities play an important role in the prognosis of COPD and greatly contribute to disease severity [2].

Depression is amongst the most common comorbidities in COPD, with an estimated prevalence ranging between 10% and 57% [3], due to different sample size, age group and depression screening tool [4]. Anyway, the diagnostic procedure is complicated by an overlap or close association of somatic and psychiatric symptoms in COPD patients who suffer from comorbid depression [5]. As a result, previous studies have reported that only 27–33% of COPD patients with depression are being treated for it [3,5,6]. The problem is serious, since the presence of depression in COPD has been associated with impaired quality of life, poor treatment adherence and increased risk of both COPD exacerbation and mortality [7,8,9]. The mechanism underlying these effects is unknown but is likely to be multifactorial [10]. Thus, greater effort should be made in identifying and managing these conditions, since early detection of depression followed by proper interventions in COPD patients could help to control depressive symptoms and clinical prognosis in COPD patients [11].

Risk factors associated with increased prevalence of this mental disorder in COPD patients are likely multiple and include age, smoking and COPD severity [2]. Female sex has also been identified as a major risk factor for depressive symptoms in adults with COPD. Moreover, the risk factors of depressive symptoms in COPD patients could differ according to sex [12].

There has been relatively little focus on depression in COPD patients, except for prevalence studies. In fact, there are hardly any studies that have evaluated the evolution of the prevalence of COPD and depression over time [13], both globally and by sex.

The objective of our study was to describe trends in the prevalence of depression in men and women with COPD hospitalized in Spain between 2016 and 2020, using a nationwide discharge database. Furthermore, we analyzed the prevalence of depression in women and men with COPD according to specific hospital admission diagnoses and hospital outcomes.

## 2. Materials and Methods

We have conducted a population-based cohort study between 1 January 2016 and 31 December 2020 using a large registry of the hospital discharges—the Spanish National Hospital Discharge Database (SNHDD)—that covers almost 100% of admissions in Spain. The methodological description of the SNHDD, as well as the coding of the variables based on the International Classification of Diseases (ICD-10) has already been described [14].

The study population included those subjects who presented a COPD code in any position of the diagnostic field of the database (see codes in Supplementary Table S1) and were ≥35 years old. To respond to the objective of the study, the study population has been stratified according to the presence of depression (see codes in Supplementary Table S1) in any position of the diagnostic field of the SNHDD. Subsequently, it has been stratified according to sex. To find out the prevalence of depression based on the most frequent hospital admission diagnoses, we used those included in the Charlson Comorbidity Index (CCI) in any position of the diagnostic field of the database, excluding COPD [15]. We also analyzed the presence of pneumonia, obesity, asthma, obstructive sleeping apnea (OSA) and the use of oxygen prior to hospital admission. Finally, the need for mechanical ventilation during hospitalization was described. Supplementary Table S1 shows the ICD-10 codes corresponding to the diagnoses and procedures used in our investigation. Regarding the outcomes of hospital admission of the subjects included in the study, we analyzed the length of hospital stay (LOHS) and in-hospital mortality (IHM).

### 2.1. Statistical Analysis

All analysis were stratified by sex. For all the years of study, in patients with COPD, we calculated the total prevalence of depression and the prevalence of depression by the main diagnoses and procedures previously described. Prevalence was calculated by dividing the number of cases of depression in each year by the number of subjects with COPD in each subgroup and year.

The descriptive statistical analysis has been carried out with the calculation of total frequencies with percentages and means with standard deviations or medians with interquartile range for categorical and continuous variables, respectively. The trend was analyzed using a Cochran–Mantel–Haenszel statistic or Cochran–Armitage test for categorical variables and linear regression t test or Jonckheere–Terpstra test for continuous variables. Categorical variables were compared using the exact Fisher test. Continuous variables were compared using the t test or the Wilcoxon rank sum test as required. We used multivariable logistic regression tests to analyze the factors associated with the presence of depression among COPD patients, evaluating the effect of sex. Finally, we used logistic regression tests to assess the effect of depression on the IHM in both men and women with COPD. Models were constructed for the concomitant clinical conditions included in the CCI, obesity and pneumonia. The results were presented using the odds ratio (OR) and 95% confidence intervals (95% CI). We used version 14 of Stata to perform the statistical analysis (Stata, College Station, TX, USA). Statistical significance was set at *p* < 0.05 (2-tailed).

### 2.2. Ethics

To carry out this study, it was not necessary to request the informed consent of the individuals nor approval by an ethics committee, since the SNHDD is anonymous and can be requested from the Spanish Ministry of Health [16].

## 3. Results

During the period between 2016 and 2020, in Spain there were 1,150,809 hospitalizations of subjects over 35 years of age who had a diagnostic code corresponding to COPD, of which 55,794 (4.85%) had a depression code. The flowchart of the study can be found in Supplementary Figure S1. The number of COPD patients rose constantly from 2016 (228,055) to 2019 (241,483), showing a marked decrement in year 2020 (195,128).

The prevalence of depression in Spain decreased significantly between 2016 and 2020 (5.09% vs. 4.68%; *p* < 0.01), as can be seen in Table 1.

Regarding the distribution of the study population, the percentage of men and women did not show significant changes over time. On the other hand, the mean age of patients with depression, as well as the associated comorbidity measured with the CCI, increased significantly throughout the study period (Table 1).

Between 2016 and 2020, depression coding as first diagnosis in the SNHDD did not change significantly (0.76% in 2016 vs. 0.56% in 2020) (Table 1).

In men with COPD, the prevalence of depression did not change significantly throughout the study period (3.42% in 2016 vs. 3.79% in 2020). The mean age (74.11 years vs. 74.70 years, *p* = 0.002) and the presence of associated comorbidity (mean CCI 2.96 vs. 3.2, *p* < 0.001) increased between 2016 and 2020. The crude IHM also increased significantly between 2016 and 2020 (6.65% vs. 9.85%, *p* < 0.001), as shown in Table 2.

In women with COPD, the prevalence of depression decreased significantly throughout the study period (12.27% in 2016 vs. 10.56% in 2020, *p* < 0.001), while mean age did not change (72.31 years vs. 72.51 years, *p* = 0.779). On the other hand, comorbidity increased significantly over time (mean CCI 2.96 vs. 3.20, *p* < 0.001). In the study period, the crude IHM also increased significantly (6.21% vs. 6.51%, *p* = 0.002) as can be seen in Table 2.

Table 3 shows the prevalence of depression by the specific hospital admission diagnoses and by mechanical ventilation during the hospitalization. Among men and women with COPD, the highest prevalence of depression was observed among those with cerebrovascular disease, liver disease, obesity, asthma, sleeping obstructive apnea and oxygen prior to hospital admission. Between 2016 and 2020, in both sexes, in those who had congestive heart failure, diabetes, obesity or OSA, the prevalence of depression decreased significantly, as well as in patients with use of oxygen prior to admission. For all years of the study and for all specific hospital admission diagnoses, the prevalence of depression was statistically higher in women with COPD than in men with COPD (*p* < 0.05).

**Table 3:** Prevalence of depression among men and women hospitalized with COPD in Spain 2016–2020, according to selected concomitant condition.

		2016	2017	2018	2019	2020	*p*-Value
Acute myocardial infarction, *n* (%)	Men	397 (2.88)	465 (2.92)	481 (2.81)	518 (2.91)	418 (2.78)	0.925
	Women	155 (9.65)	172(9.2)	181 (8.57)	196 (8.77)	198 (10.16)	0.406
Congestive heart failure, *n* (%)	Men	1347 (3.08)	1444 (3.08)	1314 (2.68)	1435 (2.89)	1079 (2.69)	<0.001
	Women	1324 (11.48)	1332 (10.85)	1153 (9.03)	1243 (9.41)	995 (9.31)	<0.001
Peripheral vascular disease, *n* (%)	Men	635 (3.19)	732 (3.35)	614 (2.8)	705 (3.05)	581 (2.92)	0.008
	Women	208 (12.11)	209 (11.58)	207 (10.51)	209 (9.19)	218 (10.4)	0.029
Cerebrovascular disease, *n* (%)	Men	603 (4.51)	628 (4.3)	532 (3.64)	615 (4.1)	479 (3.73)	0.001
	Women	300 (12.2)	349 (13.1)	312 (10.91)	333 (11.26)	331 (12.72)	0.058
Diabetes, *n* (%)	Men	1911 (3.27)	2085 (3.27)	1914 (2.9)	2044 (3.1)	1696 (3.13)	<0.001
	Women	1465 (12.26)	1611 (12.25)	1342 (10.15)	1408 (10.35)	1119 (10.37)	<0.001
Renal disease, *n* (%)	Men	1115 (3.16)	1220 (3.12)	1092 (2.67)	1234 (2.98)	943 (2.69)	<0.001
	Women	621 (9.59)	746 (10.12)	671 (8.76)	715 (8.95)	608 (9.23)	0.035
Cancer, *n* (%)	Men	1007 (2.98)	1041 (2.93)	950 (2.61)	981 (2.65)	817 (2.61)	0.002
	Women	360 (9.36)	450 (10.07)	456 (9.29)	460 (8.86)	441 (9.26)	0.371
Liver disease, *n* (%)	Men	586 (4.13)	712 (4.54)	653 (3.8)	665 (3.78)	569 (3.78)	0.001
	Women	332 (14.91)	381 (14.34)	375 (12.53)	410 (11.86)	369 (12.92)	0.004
Pneumonia, *n* (%)	Men	729 (3.47)	768 (3.44)	757 (3.33)	735 (3.4)	529 (3.29)	0.859
	Women	491 (12.65)	533 (12.53)	484 (10.38)	469 (10.16)	346 (10.42)	<0.001
Obesity, *n* (%)	Men	840 (4.1)	840 (3.64)	871 (3.64)	833 (3.37)	703 (3.32)	<0.001
	Women	1196 (15.13)	1333 (15.01)	1189 (12.83)	1197 (12.41)	978 (12.55)	<0.001
Asthma, *n* (%)	Men	106 (3.64)	143 (3.72)	153 (3.3)	186 (4.06)	172 (4.13)	0.240
	Women	393 (11.09)	490 (11.34)	561 (10.71)	584 (11.26)	404 (10.73)	0.809
Obstructive sleep apnea, *n* (%)	Men	964 (4.19)	1045 (3.95)	1037 (3.68)	1004 (3.44)	814 (3.33)	<0.001
	Women	651 (15.97)	682 (14.18)	590 (11.51)	656 (12.04)	538 (12.2)	<0.001
Oxygen prior to hospital admission, *n* (%)	Men	919 (4.2)	915 (3.89)	907 (3.77)	868 (3.68)	583 (3.44)	0.002
	Women	752 (13.33)	828 (13.29)	795 (11.47)	848 (11.63)	654 (12.14)	0.001
Mechanical ventilation, *n* (%)	Men	175 (2.75)	213 (3.05)	260 (3.15)	271 (3.25)	199 (2.64)	0.122
	Women	199 (11.72)	248 (12.08)	340 (12.77)	315 (11.01)	243 (10.73)	0.155

*p*-value for time trend. The IHM in women with depression and certain concomitant conditions, such as acute myocardial infarction, cerebrovascular disease, renal disease, liver disease and OSA, was significantly lower than in women without depression. The IHM in men with depression and most of concomitant conditions evaluated was also significantly lower than in men without depression, as shown in Table 4.

Table 5 presents the results of the multivariable analysis to identify the factors associated with the presence of depression and IHM in men and women with COPD. Older age and the presence of comorbidity acted as protective factors for the presence of depression, as well as the most recent years of hospital admission (years 2018, 2019 and 2020). The presence of obesity, OSA and the use of oxygen prior to admission were risk factors for the presenting a depression code in both men and women. The adjusted prevalence of depression was 3.54-times higher in women with COPD than in men (OR 3.54; 95%CI 3.48–3.6).

Older age, the existence of comorbidity (CCI), the presence of pneumonia, the use of oxygen prior to admission and having been hospitalized in 2020 increased the risk of IHM in men and women with COPD and depression, as indicated in Table 5. However, obesity was a protective factor, as well as OSA, for IHM in both men and women. Female sex was associated with a lower IHM in patients with COPD and depression.

Finally, in men with COPD and selected concomitant conditions such as diabetes, cancer and use of mechanical ventilation, the presence of depression decreased the risk of dying in the hospital (Supplementary Table S2). In women with COPD and almost all-specific hospital admission (except for acute myocardial infarction and asthma), the presence of depression also decreased the risk of IHM, as indicated in Supplementary Table S3.

## 4. Discussion

To the best of our knowledge, this is the first study to explore trends in the prevalence of depression in men and women hospitalized with COPD. We found that this prevalence has decreased over time in women with COPD and has not changed significantly in men with this disease. Even so, it is still more than three times higher in women than in men. Previous studies have demonstrated that depression, as well as other psychological disorders including anxiety and irritability, are more prevalent in women [17,18,19,20]. There are several sex-specific factors that could explain these results. Women are more health conscious than men and more likely to seek medical care during irregularities in their well-being [21]. In addition, they experience more self-devaluation and generally express their emotions verbally, while men turn to alcohol and other substances as a means of dealing with stress [22,23].

In relation to the determinants of the prevalence of depression in COPD patients, we detected that obesity and OSA were associated with a higher probability. Ghaemi Kerahrodi et al. also found that obesity was associated with an increase of depression in obstructive lung disease, in addition to other factors such as being a woman, smoking, low socioeconomic status, degree of dyspnea and lower pulmonary function [24]. However, Choi et al. reported that lower body mass index was a significant risk factor of depressive symptoms but only in men, not in women [12]. On the other hand, it has been reported that depression is more common in patients with overlap syndrome (COPD and OSA) than in those with pure COPD [25,26]. In this way, Heinzer et al. found that an apnea–hypopnea index > 20.6 events per hour of sleep was independently associated with the presence of depression [27]. It must be considered that mental disorders share common symptoms with both COPD and OSA, including dyspnea, chest discomfort, fatigue, sleep disturbances, loss of appetite, reduced physical activity and loss of a sense of hope, which could interfere with their diagnosis [25]. Anyway, the prevalence of depression decreased during the study period in COPD patients who had concomitant obesity or OSA, as well as congestive heart failure or use of oxygen prior to admission.

The current results showed that older age and higher comorbidity were protective factors of the presence of depression in our study population. Taking into account that older people and/or those with more comorbidities have a higher risk of mortality and that the duration of the study was 5 years, the participants in the first year could have died by the end of the fifth year. It is commonly known that the annual number of COPD cases (prevalence) comprises both new and old cases.

Regarding age, previous studies have shown that younger COPD patients are at a higher risk for depression than the elderly [18,28,29,30,31]. A number of explanations have been proposed for this finding, most focusing on the possibility that depression is underestimated among the elderly [32]. On the other hand, Tsai et al. reported that most of the young COPD patients were employed, so their workload could contribute to additional emotional distress [33].

A controversial issue is the influence of the number of comorbidities since, a priori, it would be plausible to think that the greater the number, the greater the possibility of depression, as it has been reported by other authors [34]. However, our results suggest that CCI influenced in the opposite direction: the higher the number, the less possibility of depression. In this way, the presence of concomitant comorbidities is thought to impede the recognition of depression by primary care providers [35]. However, in a more recent systematic review, Menenar et al. suggested that comorbidity burden does not consistently affect depression recognition negatively in this care level [36]. Instead, recognition seems to vary depending on the specific conditions or combination of conditions examined [36]. Along with the influence of age, CCI is another factor that deserves to be investigated in depth in the COPD–depression relationship.

In relation to mortality, our findings indicate that the crude IHM increased over time, both in men and women with COPD and depression, being higher in the former. Risk factors for IHM in our study were older age, the presence of pneumonia, a higher comorbidity and the use of oxygen prior to admission. These findings could relate to known risk factors for mortality in COPD [37]. IHM was also higher in the year 2020, probably related to the COVID-19 pandemic, since it has been shown that the presence of COPD is associated with a higher mortality from COVID-19, even after adjusting for other comorbidities [38]. On the other hand, both obesity and OSA were protective factors for IHM. In both community-dwelling and hospitalized COPD patients, several studies have reported a significant protective effect of obesity on all-cause mortality, a phenomenon known as the paradox of obesity [39]. Furthermore, chronic intermittent hypoxia caused by OSA in obese patients might be one of the underlying mechanisms in mortality paradox of obesity [40].

A surprising result of our study was that men and women with COPD who also suffered from depression had a similar or even lower risk of dying in the hospital than men and women without depression. In the same way, Martinez-Gestoso et al. found no relationship between anxiety and depression with mortality in patients admitted for COPD exacerbation, but surprisingly, mortality at 18 months was lower in patients with depression [41]. However, these findings stand in apparent contrast to previously reported associations between depression and mortality in COPD patients [42,43]. In any case, our results could also be indicative of a “depression paradox” wherein the diagnosis of depression offers an apparent protective benefit against in-hospital mortality in COPD patients. Previous studies have documented similar findings among hospitalized patients with ST-elevation myocardial infarction or breast cancer patients, also using data from a Nationwide Inpatient Sample [44,45]. One of the reasons that may explain this finding is an under-diagnosis of mental health problems among hospitalized patients with such diseases [44,45]. Another possible reason is that COPD patients with depression are hospitalized with less severe acute or chronic conditions, thus increasing their probability of survival. Regardless of whether information or selection bias are responsible for this association, further studies are required to understand the factors behind this paradox.

The major strength of this study is the large number of patients included in the analysis, providing a representative sample of patients with COPD at a national level and, thus, offering high external validity and generalizability. However, several limitations in this study should be mentioned. It is an observational, uncontrolled study based on the diagnoses described in the international code of the disease. The main cause of hospitalization of these patients is not well established and the diagnosis of depression may be underestimated because it is an additional diagnosis and not necessarily the reason for hospitalization. There was no active search for a diagnosis of depression. In addition, measurements and disease assessments were limited to the information available in the database. Thus, we were unable to determine the severity of COPD or depression or the treatment for both diseases. We also did not have information on other variables such as tobacco consumption, social relationships and social or education level, which may be related to depression [46]. Another limitation of the study was that in 2020, due to the COVID-19 pandemic and overcrowding, the diagnosis of depression in the face of the chaotic situation may have been even more underestimated. Thus, the reduction in the incidence of depression may just be underestimated and not reflect the drop itself. Finally, as we received the database completely anonymized, it is not possible to identify patients who were hospitalized more than once a year and therefore would be counted more than one time for determining the overall prevalence. Future investigations on the relationships between COPD and depression should take these aspects into account.

## 5. Conclusions

In conclusion, this study addressed knowledge gaps regarding sex differences in the trends of depression in COPD using a large sample of hospitalized patients. Our findings suggest that prevalence of depression has decreased over time in women with COPD while it has not changed significantly in men with this disease. Despite it, such prevalence is still more than three times higher in women than in men. Regarding mortality, crude IHM increased over time, both in men and women with COPD and depression, being higher in the former. Finally, we found that men and women with COPD who also suffered from depression had a similar or even lower risk of dying in the hospital than those without depression, which may be indicative of a phenomenon known as “depression paradox”. These results call for future studies to delve into the relationship between COPD and depression and evaluate the effect of specific interventions in these patients.

## Figures and Tables

**Table 1:** Characteristics of patients with COPD suffering depression according to year from 2016 to 2020.

	2016	2017	2018	2019	2020	*p*-Value Trend
COPD patients, *n*	228,055	239,767	246,376	241,483	195,128	
Depression, *n*	11,608	12,372	11,284	11,398	9132	
Depression, prevalence	5.09	5.16	4.58	4.72	4.68	<0.001
Men, *n* (%)	6326 (54.5)	6675 (53.95)	6134 (54.36)	6263 (54.95)	4975 (54.48)	0.659
Women, *n* (%)	5282 (45.5)	5697 (46.05)	5150 (45.64)	5135 (45.05)	4157 (45.52)	
Age, mean (SD)	73.29 (11.53)	73.35 (11.56)	73.24 (11.45)	73.67 (11.58)	73.70 (11.57)	0.004
40–59 years, *n* (%)	1646 (7.68)	1739 (7.88)	1510 (6.45)	1498 (6.61)	1194 (6.72)	<0.001
60–69 years, *n* (%)	2581 (5.95)	2805 (6.14)	2676 (5.5)	2589 (5.44)	2093 (5.35)	<0.001
70–79 years, *n* (%)	3283 (4.55)	3474 (4.64)	3157 (4.1)	3209 (4.13)	2622 (4.16)	<0.001
≥80 years, *n* (%)	4098 (4.51)	4354 (4.49)	3941 (4.06)	4102 (4.38)	3223 (4.28)	<0.001
CCI, mean (SD)	2.69 (1.84)	2.74 (1.87)	2.73 (1.86)	2.86 (1.94)	2.97 (2.02)	<0.001
Depression first diagnosis, *n* (%)	88 (0.76)	83 (0.67)	71 (0.63)	58 (0.51)	51 (0.56)	0.147
LOHS, median (IQR)	6 (7)	6 (8)	6 (6)	6 (7)	6 (8)	0.982
IHM, *n* (%)	695 (5.99)	764 (6.18)	702 (6.22)	724 (6.35)	762 (8.34)	<0.001

CCI—Charlson Comorbidity Index; LOHS—length of hospital stay; IHM—in-hospital mortality.

**Table 2:** Prevalence of depression, age distribution, clinical characteristics and in-hospital outcomes among hospitalized men and women with COPD in Spain 2016–2020.

		2016	2017	2018	2019	2020	*p*-Value
Men	Depression, *n* (Prevalence)	6326 (3.42)	6675 (3.45)	6134 (3.11)	6263 (3.25)	4975 (3.19)	0.274
	Age, mean (SD)	74.11 (10.82)	74.27 (11)	74.05 (10.93)	74.64 (11.12)	74.7 (11.22)	0.002
	40–59 years, *n* (%)	688 (4.69)	769 (5.09)	687 (4.28)	686 (4.45)	548 (4.47)	<0.001
	60–69 years, *n* (%)	1387 (3.96)	1388 (3.85)	1322 (3.51)	1265 (3.49)	986 (3.31)	<0.001
	70–79 years, *n* (%)	1927 (3.12)	2029 (3.19)	1864 (2.87)	1886 (2.9)	1564 (2.95)	<0.001
	≥80 years, *n* (%)	2324 (3.16)	2489 (3.17)	2261 (2.87)	2426 (3.2)	1877 (3.08)	<0.001
	CCI, mean (SD)	2.96 (1.97)	3.01 (1.99)	2.98 (1.93)	3.14 (2.06)	3.2 (2.09)	<0.001
	Depression first diagnosis, *n* (%)	48 (0.76)	58 (0.87)	48 (0.78)	35 (0.56)	32 (0.64)	0.273
	LOHS, median (IQR)	6 (7)	6 (8)	6 (7)	6 (7)	6 (8)	0.897
	IHM, *n* (%)	420 (6.64)	478 (7.16)	448 (7.3)	473 (7.55)	490 (9.85)	<0.001
Women	Depression, *n* (Prevalence)	5282 (12.27)	5697 (12.28)	5150 (10.58)	5135 (10.46)	4157 (10.56)	<0.001
	Age, mean (SD)	72.31 (12.25)	72.28 (12.1)	72.26 (11.96)	72.49 (12.02)	72.49 (11.87)	0.779
	40–59 years, *n* (%)	958 (14.21)	970 (13.95)	823 (11.14)	812 (11.21)	646 (11.72)	<0.001
	60–69 years, *n* (%)	1194 (14.26)	1417 (14.62)	1354 (12.3)	1324 (11.65)	1107 (11.87)	<0.001
	70–79 years, *n* (%)	1356 (12.88)	1445 (12.87)	1293 (10.83)	1323 (10.47)	1058 (10.51)	<0.001
	≥80 years, *n* (%)	1774 (10.2)	1865 (10.07)	1680 (9.16)	1676 (9.38)	1346 (9.31)	<0.001
	CCI, mean (SD)	2.38 (1.61)	2.43 (1.67)	2.44 (1.73)	2.52 (1.73)	2.7 (1.89)	<0.001
	Depression first diagnosis, *n* (%)	40 (0.76)	25 (0.44)	23 (0.45)	23 (0.45)	19 (0.46)	0.094
	LOHS, median (IQR)	6 (7)	6 (7)	6 (6)	6 (6)	6 (7)	0.553
	IHM, *n* (%)	275 (5.21)	286 (5.02)	254 (4.93)	251 (4.89)	272 (6.54)	0.002

CCI—Charlson Comorbidity Index; Prevalence—prevalence of depression among patients with COPD; LOH—length of hospital stay; IHM—in-hospital mortality.

**Table 4:** In-hospital mortality among men and women hospitalized with COPD in Spain 2016–2020, according to selected concomitant condition and to the presence of depression.

	IHM MEN			IHM WOMEN		
	Without Depression	With Depression	*p*-Value	Without Depression	With Depression	*p*-Value
Acute myocardial infarction, *n* (%)	7037 (9.1)	177 (7.77)	0.029	856 (9.65)	71 (7.87)	0.082
Congestive heart failure, *n* (%)	25,250 (11.33)	708 (10.7)	0.111	6262 (11.5)	499 (8.25)	<0.001
Peripheral vascular disease, *n* (%)	8976 (8.68)	258 (7.9)	0.117	817 (9.27)	72 (6.85)	0.010
Cerebrovascular disease, *n* (%)	8479 (12.56)	313 (10.96)	0.011	1624 (13.63)	153 (9.42)	<0.001
Diabetes, *n* (%)	24,156 (8.09)	734 (7.61)	0.088	4432 (7.95)	382 (5.5)	<0.001
Renal disease, *n* (%)	21,182 (11.37)	548 (9.78)	<0.001	3852 (11.77)	276 (8.21)	<0.001
Cancer, *n* (%)	24,239 (14.32)	678 (14.14)	0.725	2887 (13.74)	245 (11.31)	0.002
Liver disease, *n* (%)	6912 (9.03)	192 (6.03)	<0.001	902 (7.32)	95 (5.09)	<0.001
Pneumonia, *n* (%)	12,043 (12.02)	406 (11.54)	0.391	1833 (9.96)	170 (7.32)	<0.001
Obesity, *n* (%)	6477 (5.93)	225 (5.51)	0.261	2287 (6.08)	274 (4.65)	<0.001
Asthma, *n* (%)	1120 (5.78)	47 (6.18)	0.639	1163 (5.93)	122 (5.02)	0.071
Obstructive sleep apnea, *n* (%)	7797 (6.16)	246 (5.06)	0.002	1195 (5.76)	121 (3.88)	<0.001
Oxygen prior to hospital admission, *n* (%)	11,460 (10.84)	388 (9.26)	0.001	2747 (9.95)	298 (7.69)	<0.001
Mechanical ventilation, *n* (%)	9562 (26.3)	247 (22.09)	0.002	2077 (20.37)	184 (13.68)	<0.001

IHM—in-hospital mortality. *p*-value for the comparison between those with and without depression.

**Table 5:** Multivariate analysis of the factors associated with the presence of depression among men and women hospitalized with COPD and in-hospital mortality among patients with COPD and concomitant depression, Spain 2011–2020.

	Presence of Depression			IHM among Patients with COPD and Depression		
	Men	Women	Both Sexes	Men	Women	Both Sexes
	OR (95%CI)	OR (95%CI)	OR (95%CI)	OR (95%CI)	OR (95%CI)	OR (95%CI)
Year 2016	1	1	1	1	1	1
Year 2017	1.01 (0.97–1.05)	1 (0.96–1.04)	1.01 (0.98–1.03)	1.07 (0.93–1.23)	0.94 (0.79–1.12)	1.02 (0.91–1.13)
Year 2018	0.9 (0.87–0.94)	0.84 (0.81–0.87)	0.88 (0.85–0.9)	1.07 (0.92–1.23)	0.86 (0.72–1.02)	0.98 (0.88–1.1)
Year2019	0.95 (0.92–0.99)	0.83 (0.79–0.86)	0.9 (0.87–0.92)	1.05 (0.91–1.21)	0.83 (0.69–1)	0.96 (0.86–1.07)
Year 2020	0.94 (0.9–0.97)	0.84 (0.81–0.88)	0.9 (0.87–0.92)	1.43 (1.25–1.65)	1.13 (0.94–1.35)	1.31 (1.17–1.46)
Age, 40–59 years	1	1	1	1	1	1
Age, 60–69 years	0.78 (0.75–0.82)	1.04 (1–1.09)	0.91 (0.89–0.94)	1.65 (1.32–2.06)	1.36 (1.06–1.74)	1.52 (1.29–1.79)
Age, 70–79 years	0.65 (0.62–0.67)	0.92 (0.88–0.96)	0.77 (0.74–0.79)	2.32 (1.88–2.86)	2.15 (1.7–2.71)	2.26 (1.93–2.63)
Age, ≥80 years	0.67 (0.64–0.69)	0.77 (0.74–0.8)	0.73 (0.71–0.75)	3.85 (3.14–4.72)	4.21 (3.39–5.22)	3.99 (3.44–4.63)
CCI	0.97 (0.97–0.98)	0.95 (0.95–0.96)	0.9 6(0.96–0.97)	1.2 6(1.24–1.29)	1.31 (1.27–1.34)	1.28 (1.26–1.3)
Pneumonia	1.05 (1.01–1.09)	1.01 (0.97–1.06)	1.04 (1.01–1.07)	1.57 (1.4–1.77)	1.41 (1.19–1.68)	1.52 (1.38–1.67)
Obesity	1.04 (1–1.08)	1.27 (1.23–1.31)	1.16 (1.13–1.19)	0.76 (0.65–0.88)	0.86 (0.74–0.99)	0.81 (0.73–0.89)
Asthma	1.11 (1.03–1.2)	0.97 (0.93–1.02)	1.01 (0.97–1.04)	0.95 (0.7–1.3)	0.98 (0.8–1.19)	0.97 (0.82–1.15)
Obstructive sleep apnea	1.12 (1.08–1.16)	1.08 (1.04–1.13)	1.1 (1.07–1.13)	0.67 (0.58–0.77)	0.67 (0.55–0.82)	0.67 (0.6–0.76)
Oxygen prior to hospital admission	1.21 (1.17–1.25)	1.13 (1.09–1.17)	1.17 (1.14–1.2)	1.27 (1.12–1.43)	1.48 (1.29–1.7)	1.35 (1.23–1.48)
Mechanical ventilation	0.84 (0.79–0.9)	0.95 (0.9–1.01)	0.9 (0.86–0.94)	5.17 (4.41–6.06)	4.51 (3.77–5.4)	4.84 (4.3–5.45)
Female			3.54 (3.48–3.6)			0.81 (0.76–0.88)

CCI—Charlson Comorbidity Index; IHM—in-hospital mortality; OR—odds ratio; CI—confidence interval.

## Data Availability

According to the contract signed with the Spanish Ministry of Health and Social Services, which provided access to the databases from the Spanish National Hospital Database (SNHDD), we cannot share the databases with any other investigator and we have to destroy the databases once the investigation has concluded. Consequently, we cannot upload the databases to any public repository. However, any investigator can apply for access to the databases by filling out the questionnaire available at https://www.sanidad.gob.es/estadEstudios/estadisticas/estadisticas/estMinisterio/SolicitudCMBD.htm (accessed on 24 October 2022). All other relevant data are included in the paper.

## Supplementary Materials

The following supporting information can be downloaded at: https://www.mdpi.com/article/10.3390/jcm11216337/s1, Figure S1. Flowchart of COPD patient’s selection and hospital outcome according to the presence of depression and sex; Table S1: Diagnosis and procedures analyzed with their corresponding ICD10 codes; Table S2: Multivariate analysis of the factors associated with IHM in men with COPD and selected concomitant conditions in Spain, 2011–2020; Table S3: Multivariate analysis of the factors associated with IHM in women with COPD and selected concomitant conditions in Spain 2011–2020.
