# Supplementary material for: National Trends in Prevalence of Depression in Men and Women with Chronic Obstructive Pulmonary Disease Hospitalized in Spain, 2016–2020
Source: J Clin Med. 2022 Oct 27;11(21):6337. doi: 10.3390/jcm11216337 (PMC9655616; doi:10.3390/jcm11216337)
Supplement: Supplementary file 1 [file jcm-11-06337-s001.zip › jcm-1957432-supplementary.pdf]

Table S1. Diagnosis and procedures analyzed with their corresponding ICD10 codes.

DIAGNOSIS	ICD-10 codes
COPD	I27.8, I27.9, J40.x–J47.x, J60.x–J67.x, J68.4, J70.1, J70.3
Depression	F34.1, F43.21; F32.9
Acute Myocardial Infarction	I21, I22, I252
Congestive Heart Failure	I50
Peripheral Vascular Disease	I71, I790, I739, R02, Z958, Z959
Cerebrovascular Disease	I60, I61, I62, I63, I65, I66,G450, G451, G452, G458, G459, G46, I64, G454, I670, I671, I672, I674, I675, I676, I677 I678, I679, I681, I682, I688, I69
Type 2 diabetes	E11.x
Renal Disease	I12.0, I13.1, N03.2–N03.7, N05.2–N05.7, N18.x, N19.x, N25.0, Z49.0–Z49.2, Z94.0, Z99.2
Liver Disease	B18.x, K70.0-K70.3, K70.9, K71.3-K71.5, K71.7, K73.x, K74.x, K76.0, K76.2-K76.4, K76.8, K76.9, Z94.4, I85.0, I85.9, I86.4, I98.2, K70.4, K71.1, K72.1, K72.9, K76.5, K76.6, K76.7
Cancer	C00.x-C26.x, C30.x-C34.x, C37.x-C41.x, C43.x, C45.x-C58.x, C60.x-C76.x, C81.x-C85.x, C88.x, C90.x-C97.x, C77.x-C80.x
Obesity	E66.09, E66.1 E66.3, E66.8 E66.9 E66.2, E66.01
Pneumonia	J12 to J18¸ J12-J18, J95.851
Asthma	J45
Obstructive sleep apnea	G47.33
Oxygen prior to hospital admission	Z99.81
Mechanical ventilation	5A1945Z, 5A1955Z, 5A1935Z,5A09357, 5A09457, 5A09557

Figure S1. Flowchart of COPD patient's selection and hospital outcome according to the presence of depression and sex

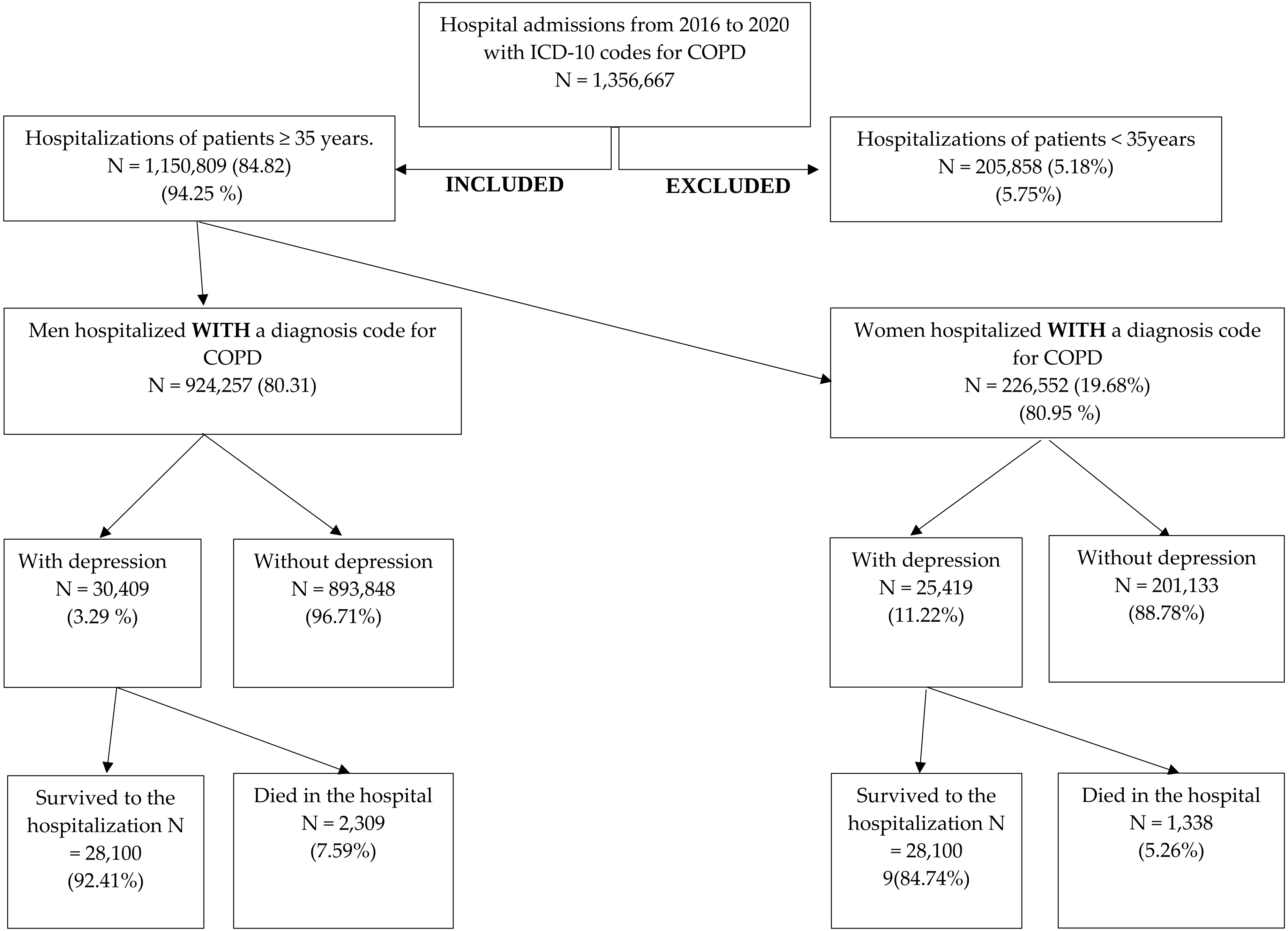

Table S2. Multivariate analysis of the factors associated with IHM in men with COPD and selected concomitant conditions in Spain, 2016-2020.

	Acute myocardial infarction	Congestive heart failure	Peripheral vascular disease	Cerebrovascular disease	Diabetes	Renal disease	Cancer	Liver disease	Pneumonia	Obesity	Asthma	Sleeping obstructive apnea	Oxygen prior to hospital admission	Mechanical ventilation
	OR(95%CI)	OR(95%CI)	OR(95%CI)	OR(95%CI)	OR(95%CI)	OR(95%CI)	OR(95%CI)	OR(95%CI)	OR(95%CI)	OR(95%CI)	OR(95%CI)	OR(95%CI)	OR(95%CI)	OR(95%CI)
Year 2016	1	1	1	1	1	1	1	1	1	1	1	1	1	1
Year 2017	0.98(0.9–1.06)	0.99(0.95–1.03)	1.03(0.96–1.1)	0.98(0.91–1.06)	0.98(0.94–1.02)	1.03(0.98–1.08)	0.98(0.94–1.02)	0.91(0.84–0.99)	0.99(0.93–1.05)	0.99(0.91–1.08)	1.03(0.82–1.3)	1.01(0.93–1.09)	0.96(0.9–1.02)	0.96(0.89–1.04)
Year 2018	0.99(0.91–1.07)	1.02(0.98–1.06)	1.02(0.95–1.1)	1(0.93–1.07)	0.97(0.93–1.01)	1.03(0.98–1.08)	0.97(0.93–1.01)	0.93(0.85–1)	0.99(0.94–1.05)	0.99(0.91–1.08)	1.27(1.03–1.57)	1.05(0.97–1.13)	1(0.94–1.06)	0.87(0.81–0.94)
Year2019	0.94(0.87–1.02)	0.99(0.95–1.04)	0.96(0.89–1.03)	0.94(0.87–1.01)	0.93(0.89–0.97)	0.99(0.94–1.03)	0.92(0.88–0.96)	0.91(0.84–0.98)	0.92(0.87–0.98)	0.96(0.89–1.05)	0.99(0.8–1.24)	1.01(0.93–1.09)	0.97(0.91–1.03)	0.82(0.76–0.89)
Year 2020	1.19(1.1–1.29)	1.24(1.19–1.29)	1.18(1.1–1.27)	1.13(1.05–1.21)	1.21(1.16–1.27)	1.24(1.18–1.3)	1.03(0.99–1.08)	1.08(1–1.17)	1.19(1.12–1.26)	1.44(1.33–1.56)	1.52(1.23–1.87)	1.49(1.39–1.61)	1.24(1.17–1.32)	1.2(1.11–1.29)
Age, 40-59 years	1	1	1	1	1	1	1	1	1	1	1	1	1	1
Age, 60-69 years	1.3(1.1–1.54)	1.33(1.2–1.48)	1.07(0.93–1.25)	1.09(0.93–1.27)	1.34(1.23–1.47)	1.25(1.1–1.43)	1.03(0.97–1.1)	1.5(1.38–1.63)	1.41(1.26–1.59)	1.3(1.16–1.46)	1.9(1.38–2.63)	1.29(1.15–1.45)	1.63(1.42–1.86)	1.42(1.29–1.56)
Age, 70-79 years	1.86(1.59–2.18)	1.76(1.6–1.94)	1.44(1.25–1.66)	1.4(1.21–1.62)	1.76(1.62–1.91)	1.67(1.47–1.89)	1.12(1.05–1.19)	1.75(1.62–1.9)	1.68(1.51–1.87)	1.77(1.59–1.98)	2.47(1.84–3.31)	1.76(1.57–1.96)	1.98(1.74–2.24)	1.95(1.78–2.13)
Age, ≥80 years	3.32(2.84–3.88)	2.97(2.7–3.26)	2.57(2.23–2.95)	2.24(1.94–2.59)	3.08(2.84–3.34)	2.96(2.62–3.35)	1.56(1.47–1.66)	2.59(2.38–2.81)	2.51(2.26–2.78)	3.2(2.87–3.57)	5.09(3.84–6.74)	3.11(2.78–3.47)	3.07(2.71-3.47)	2.42(2.21–2.65)
CCI	1.22(1.21–1.23)	1.16(1.15–1.16)	1.21(1.19–1.22)	1.16(1.15–1.18)	1.26(1.25–1.27)	1.2(1.19–1.21)	1.25(1.24–1.25)	1.26(1.24–1.27)	1.21(1.2–1.22)	1.25(1.24–1.26)	1.28(1.25–1.31)	1.28(1.27–1.29)	1.18(1.17–1.19)	1.13(1.12–1.15)
Depression	0.92(0.79–1.08)	0.95(0.84–1.09)	0.9(0.8–1.01)	1.01(0.94–1.09)	0.88(0.8–0.96)	1.01(0.93–1.1)	0.75(0.65–0.87)	1(0.9–1.11)	0.99(0.86–1.14)	1.26(0.93–1.72)	0.93(0.81–1.06)	0.91(0.82–1.02)	0.86(0.75–1)	0.86(0.75–0.99)

CCI: Charlson Comorbidity Index

Table S3. Multivariate analysis of the factors associated with IHM in women with COPD and selected concomitant conditions in Spain, 2016-2020.

	Acute myocardial infarction	Congestive heart failure	Peripheral vascular disease	Cerebrovascular disease	Diabetes	Renal disease	Cancer	Liver disease	Pneumonia	Obesity	Asthma	Sleeping obstructive apnea	Oxygen prior to hospital admission	Mechanical ventilation
	OR(95%CI)	OR(95%CI)	OR(95%CI)	OR(95%CI)	OR(95%CI)	OR(95%CI)	OR(95%CI)	OR(95%CI)	OR(95%CI)	OR(95%CI)	OR(95%CI)	OR(95%CI)	OR(95%CI)	OR(95%CI)
Year 2016	1	1	1	1	1	1	1	1	1	1	1	1	1	1
Year 2017	1(0.8–1.26)	0.95(0.88–1.03)	0.97(0.77–1.22)	0.99(0.84–1.16)	1.02(0.93–1.13)	1.02(0.92–1.14)	0.97(0.85–1.11)	1.02(0.82–1.27)	0.95(0.82–1.1)	0.91(0.8–1.04)	1.03(0.84-1.26)	1.22(1.01–1.49)	0.9(0.8–1.02)	1.03(0.88–1.21)
Year 2018	0.83(0.66–1.04)	0.96(0.89–1.04)	0.88(0.7–1.11)	0.98(0.84–1.15)	0.96(0.87–1.06)	1(0.9–1.12)	0.89(0.79–1.02)	0.86(0.69–1.07)	0.86(0.74–1)	0.91(0.8–1.04)	0.95(0.79-1.16)	1.07(0.88–1.31)	0.94(0.83–1.06)	0.78(0.67–0.92)
Year2019	0.9(0.72–1.12)	0.93(0.85–1.01)	0.85(0.68–1.06)	0.94(0.8–1.11)	0.96(0.87–1.05)	1.05(0.94–1.16)	0.98(0.86–1.11)	0.78(0.62–0.96)	0.75(0.64–0.87)	0.92(0.81–1.05)	1.03(0.85-1.25)	1.12(0.93–1.36)	0.9(0.8–1.02)	0.82(0.7–0.95)
Year 2020	1.06(0.84–1.32)	1.18(1.09–1.28)	1.04(0.83–1.29)	1.06(0.9–1.25)	1.24(1.12–1.36)	1.21(1.09–1.35)	1(0.88–1.14)	1.03(0.83–1.28)	1.1(0.95–1.29)	1.3(1.14–1.48)	1.4(1.15-1.7)	1.65(1.37–1.99)	1.18(1.04–1.33)	1.02(0.87–1.19)
Age, 40-59 years	1	1	1	1	1	1	1	1	1	1	1	1	1	1
Age, 60-69 years	2.51(1.6–3.94)	1.51(1.22–1.86)	1.88(1.26–2.81)	1.32(1–1.74)	1.38(1.17–1.63)	1.31(1.01–1.7)	1.18(1.05–1.34)	1.95(1.59–2.39)	1.55(1.23–1.96)	1.61(1.33–1.95)	1.41(1.04-1.89)	1.67(1.27–2.2)	1.78(1.44–2.2)	1.32(1.13–1.55)
Age, 70-79 years	2.99(1.92–4.64)	1.92(1.58–2.34)	2.3(1.56–3.41)	1.42(1.09–1.84)	1.88(1.61–2.21)	1.88(1.49–2.38)	1.3(1.15–1.48)	2.56(2.09–3.14)	2.51(2.03–3.11)	2.39(2–2.86)	2.18(1.67-2.85)	2.75(2.13–3.56)	2.27(1.86–2.78)	2(1.72–2.32)
Age, ≥80 years	6.01(3.93–9.19)	3.45(2.86–4.17)	4.17(2.85–6.09)	2.6(2.03–3.33)	3.69(3.18–4.29)	3.25(2.59–4.07)	1.95(1.73–2.21)	3.66(3.01–4.45)	4.14(3.39–5.05)	4.34(3.65–5.15)	4.68(3.68-5.95)	4.51(3.51–5.8)	4.1(3.39–4.95)	2.26(1.93–2.64)
CCI	1.19(1.15–1.24)	1.17(1.16–1.19)	1.21(1.17–1.26)	1.17(1.14–1.2)	1.28(1.27–1.3)	1.21(1.18–1.23)	1.28(1.26–1.3)	1.29(1.26–1.32)	1.23(1.2–1.26)	1.26(1.23–1.28)	1.32(1.29-1.36)	1.26(1.23–1.3)	1.18(1.15–1.2)	1.19(1.16–1.22)
Depression	0.85(0.66–1.1)	0.73(0.66–0.8)	0.77(0.6–0.99)	0.69(0.58–0.82)	0.76(0.69–0.85)	0.71(0.63–0.81)	0.84(0.72–0.96)	0.75(0.6–0.94)	0.78(0.66–0.92)	0.86(0.76–0.98)	0.94(0.77-1.14)	0.76(0.62–0.92)	0.85(0.75–0.96)	0.68(0.58–0.8)

CCI: Charlson Comorbidity Index
